# Effect of a digital sexual violence prevention education program on digital sexual violence prevention knowledge, self-protection behavior, and digital citizenship among fifth-grade children: a quasi-experimental study

**DOI:** 10.4069/whn.2025.12.08.1

**Published:** 2025-12-31

**Authors:** Miae Oh, Sukhee Ahn

**Affiliations:** 1 Jincheon Sangsan Elementary School, Jincheon, Korea; 2 College of Nursing, Chungnam National University, Daejeon, Korea

**Keywords:** Children, Digital sexual violence, Education, Health, School

## Abstract

**Purpose:**

This study aimed to develop a digital sexual violence prevention education program for upper-grade elementary school students and to examine its effects on students’ digital sexual violence prevention knowledge, self-protection behavior, and digital citizenship.

**Methods:**

A quasi-experimental pre- and posttest design was employed to evaluate the effectiveness of the program. A digital sexual violence prevention education consisting of four sessions was developed, covering core content related to digital sexual violence, digital citizenship education, and gender sensitivity. Participants were fifth-grade students from an elementary school in Cheongju, Korea. Two classes (n=27) were randomly assigned to the experimental group, and two classes (n=20) were assigned to the control group. The experimental group received one session per week for four weeks, whereas the control group received no intervention. Outcome measures included digital sexual violence prevention knowledge, self-protection behavior, and digital citizenship. A one-tailed test was applied to determine the effectiveness of the education program in producing improvement.

**Results:**

The experimental group demonstrated significantly greater digital sexual violence prevention knowledge and bystander self-efficacy compared with the control group (*p*<.05). However, no significant group differences were observed in self-protection behavior or in other domains of digital citizenship.

**Conclusion:**

The four-session digital sexual violence prevention education program effectively enhanced digital sexual violence prevention knowledge and bystander self-efficacy related to digital citizenship among fifth-grade students. Further research using a randomized controlled design with a larger sample size is recommended to confirm and extend these findings.

## Introduction

Children referred to as the digital native generation actively engage with digital environments [1]. For these children, online spaces function as venues for interacting with unfamiliar individuals and as a primary means for accessing necessary information conveniently and rapidly [2]. At the same time, the anonymity and non-face-to-face nature of online environments create conditions in which crimes that infringe upon victims’ dignity and personal rights can easily occur, rendering digital spaces potentially dangerous [3].

Digital sexual violence broadly refers to gender-based violence that occurs both online and offline through the use of digital devices and information and communication technologies. Its scope includes illegal filming conducted without consent; distribution, re-distribution, and threats of distribution against the individual’s will regardless of consent to filming; the production of synthetic content such as deepfakes; possession, purchase, or storage of such materials; and sexual harassment occurring within cyberspace [4]. According to the 2020 Ministry of Education school violence survey, the overall rate of digital sexual violence perpetration or victimization during the past year was 0.22%, with perpetration and victimization rates decreasing as school grade increased. Notably, the prevalence among elementary school students (0.3%) was higher than that among middle school (0.2%) and high school students (0.1%) [5]. Similarly, the 2021 Seoul survey on digital sexual crime victimization among children and adolescents found that upper-grade elementary school students demonstrated lower levels of awareness regarding digital sexual crime information and its seriousness compared with middle and high school students [6]. These findings indicate that children constitute the group most vulnerable to online sexual crime risk due to developmental immaturity in judgment and self-protection abilities, as well as insufficient gender sensitivity.

As smartphone and social networking service (SNS) use continues to increase in modern society, sexually explicit content spreads rapidly within digital spaces, negatively influencing the formation of sexual values and perceptions among children and adolescents who are still in critical stages of cognitive and emotional development [7]. In particular, sexual violence against children facilitated through digital media poses serious harm, as once such content is disseminated online, it spreads quickly, is difficult to remove, and is repeatedly copied and redistributed. Moreover, because complete deletion of victimization-related content is often impossible, victims’ psychological distress and social stigma persist over extended periods, amplifying the severity of the crime [1,8]. Accordingly, the prevention of digital sexual violence requires the cultivation of digital citizenship, defined as the attitudes and competencies necessary to act responsibly and ethically within digital environments [9]. Digital citizenship is essential for all digital media users and plays a central role in establishing a healthy and safe digital society.

The period during which sexual violence prevention education is most needed is the fourth to sixth grades of elementary school [10]. This is because access to sex-related media has become increasingly easy due to the widespread use of smartphones and the internet [11], because incidents of both perpetration and victimization increase from the elementary school years as a result of limited understanding of digital sexual violence, and because there is a high demand for education aimed at preventing exposure to risk and fostering a safe sexual culture within digital environments [12]. Reflecting these educational needs, the content of digital sexual violence prevention education for adolescents includes understanding personal information protection and copyright to prevent perpetration and victimization in digital environments, cultivating safe communication practices through empathy and consideration, and fostering citizenship that actively participates in the prevention of digital violence and sexual violence [13,14]. Since 2019, the Ministry of Gender Equality and Family, public institutions, women’s organizations, and provincial offices of education have produced and distributed educational materials for digital sexual violence prevention; however, there remain relatively few studies examining the application of educational programs utilizing these materials or evaluating the effectiveness of such programs.

Related studies include a research report in which the AHA Seoul Municipal Youth Sex Culture Center developed a digital sexual violence prevention education program and applied it through sex education experts to fifth–sixth-grade elementary school students (two sessions) and first–third-grade middle school students (two to three sessions), followed by verification of its educational effectiveness [13]. Elementary school students who participated in this program demonstrated increased post-education scores, regardless of gender, in areas including respect and consideration in the digital world, digital citizenship, active bystander participation, and knowledge of digital sexual violence and response strategies, thereby confirming the positive effects of the prevention education [13]. Additional analysis of the same research data revealed that the magnitude of post-education score improvement was greater among elementary school students than among middle school students, and that educational effects were lower when prior knowledge and awareness of digital sexual violence were limited [14]. Although these prior studies [13,14] demonstrated short-term effects through the application of a two-session digital sexual violence prevention education program for upper-grade elementary school students, they were limited by the use of a single-group pretest–posttest design. Accordingly, the present study seeks to develop a digital sexual violence prevention education program that is appropriate for the developmental level of upper-grade elementary school students, feasible for implementation during school health education hours, and grounded in a systematic instructional design model, and to examine the outcomes of this program using a quasi-experimental design with a control group.

The purpose of this study is to develop a digital sexual violence prevention education program for upper-grade elementary school students and to verify its educational effects through program implementation. The research hypotheses established accordingly are as follows.

• Hypothesis 1. Students who participate in the digital sexual violence prevention education program will have higher digital sexual violence prevention knowledge scores than students who do not participate.

• Hypothesis 2. Students who participate in the digital sexual violence prevention education program will have higher self-protection behavior scores for digital sexual violence prevention than students who do not participate.

• Hypothesis 3. Students who participate in the digital sexual violence prevention education program will have higher scores in each of the four subdomains of digital citizenship than students who do not participate.

## Methods

**Ethics statement:** This study was approved by the Institutional Review Board of Chungnam National University (No. 202212-SB-176-01). The study was advertised through a class letter sent home, and informed consent was obtained from parents. Students who returned the signed consent forms were subsequently asked to provide their assent to participate. An independent research assistant managed the study procedures to ensure the protection of the children’s human rights.

### Study design

This study employed a nonequivalent control group pretest–posttest design as a quasi-experimental study to analyze the effects of a digital sexual violence prevention education program on digital sexual violence prevention knowledge, self-protection behavior, and digital citizenship. Prior to this analysis, a methodological study was conducted to develop the digital sexual violence prevention education program for fifth-grade elementary school students. This study was reported in accordance with the TREND (Transparent Reporting of Evaluations with Nonrandomized Designs) guidelines for quasi-experimental research [15].

#### Digital sexual violence prevention education program development: based on the ADDIE model

The analysis, design, development, implementation, and evaluation (ADDIE) model [16] enables educational programs to be designed, implemented, and evaluated in a systematic and consistent manner. Accordingly, this study adopted the ADDIE model to logically structure the program by reflecting learners’ needs and to establish corresponding operational and evaluation plans (Appendix 1) for the digital sexual violence prevention education program.

##### 1) Analysis phase

In this study, the need for digital sexual violence prevention education was assessed, and an analysis was conducted through an examination of learners, educational environments, and a review of relevant literature. First, a survey of educational needs among upper-grade elementary school homeroom teachers indicated that demand for sex education and sexual violence prevention education was the highest. Additionally, lifestyle surveys of upper-grade elementary school students revealed that most students communicated not only at school but also in online group chats via smartphones, and that school violence incidents related to digital sexual violence—such as illegal filming using smartphones in educational settings, verbal abuse in online group chats, and the transmission of photos or videos without consent—were increasing. Review studies of sexual violence prevention education intervention programs showed that although education duration ranged from a minimum of 2 weeks to a maximum of 15 weeks, studies lasting 4 weeks or less were the most common, accounting for eight studies (36.4%), and that sexual knowledge, sexual attitudes, and sexual violence coping skills were frequently used as evaluation indicators in intervention research [17,18].

##### 2) Design phase

(1) The goal of the digital sexual violence prevention education program was to promote understanding of the concept of digital sexual violence, to cultivate knowledge and attitudes that enable self-protection in digital sexual violence situations occurring in online spaces, and to foster the ability to perform the role of an active bystander when encountering dangerous situations. (2) Educational content was extracted by focusing on the thematic areas and content of the one-session Elementary Upper-Grade Digital Sexual Crime Prevention Education materials produced and distributed by the Ministry of Gender Equality and Family and the Korean Institute for Gender Equality Promotion and Education, as well as the eight-session Gender Sensitivity Education for Digital Sexual Violence Prevention materials for elementary school students produced and distributed by the Ministry of Education. In addition, relevant prior studies and educational materials provided by various government ministries and provincial offices of education were used as reference sources. The program was structured into four sessions after considering that prior digital sexual violence prevention education programs for upper-grade elementary school students were developed and implemented as two-session programs [13], that most sexual violence prevention education programs for elementary school students consist of three to five sessions [17,18], and that school health education schedules impose practical constraints on program implementation. (3) With respect to teaching–learning methods, previous studies have shown that the use of diverse instructional methods and media positively influences the effectiveness of sexual violence prevention education programs, as elementary school students demonstrate strong learning motivation and active engagement due to their developmental characteristics [17]. Accordingly, to enhance students’ interest and educational effectiveness, this study developed instructional materials such as PowerPoint slides and activity worksheets and employed a range of teaching–learning strategies, including lectures, assignments, lessons incorporating open-ended questions, games and small-group activities, role-playing, quizzes, and hands-on activities using instructional aids. (4) For the evaluation design, pre- and post-intervention surveys of outcome variables were conducted for both the experimental and control groups. When baseline levels of outcome variables were equivalent between the groups, the effectiveness of the program was planned to be evaluated by comparing post-intervention outcome scores between groups.

##### 3) Development phase

The digital sexual violence prevention education program developed in this study was structured as a four-session curriculum delivered once per week for 40 minutes during health education classes. Based on the analyzed data and instructional considerations identified during the planning process, four session-specific lesson plans were developed by referring to activity sheets, videos, and PowerPoint materials included in the instructional resource package. Educational materials and activity worksheets were then created to align with the content and objectives of each session. The detailed content of each session within the digital sexual violence prevention education program is presented in Table 1.

##### 4) Implementation and evaluation plan phase

The research team planned the detailed implementation procedures, evaluation indicators, and evaluation methods for the developed digital sexual violence prevention education program and conducted preparatory activities necessary for its application.

#### Execution of quasi-experimental study

##### 1) Samples

The participants in this study were convenience-sampled from fifth-grade students at a single elementary school located in Cheongju, with an approximate total student enrollment of 550. Inclusion criteria were students who expressed interest in the study, understood the purpose and procedures of the research, and were able to comprehend and respond to the questionnaire. Exclusion criteria included students receiving special education who experienced difficulty understanding health education content or completing survey responses. Fifth-grade students were selected as the study population because, according to the 2022 Teenage Youth Media Usage Survey conducted by the Korea Press Foundation [19], smartphone ownership and usage rates increase sharply from 70% in fourth grade to 85% in fifth grade, marking the onset of substantial exposure to digital environments. Accordingly, it was anticipated that digital sexual violence prevention education would have the greatest effect among fifth-grade students. The first researcher instructed the four homeroom teachers of the fifth-grade classes to simultaneously and randomly draw one of four opaque, sealed envelopes containing slips labeled either “experimental group” or “control group.” As a result, the two classes that drew slips labeled “experimental group” were assigned to the experimental group (n=27) and received the digital sexual violence prevention education program, while the two classes that drew slips labeled “control group” were assigned to the control group (n=20) and received standard health education according to the existing health education schedule.

The required sample size was calculated using G*Power ver. 3.1 (Heinrich Heine University Düsseldorf, Düsseldorf, Germany) based on the effect size reported in a previous study [14]. Assuming an effect size of d=0.8, a significance level of α=0.05 (one-tailed test), and a statistical power of 0.80, the analysis indicated that 21 participants per group, for a total of 42 participants, were needed [20]. To account for an anticipated dropout rate of approximately 10% due to early dismissal, absence, school transfer, or incomplete questionnaire responses, the first researcher planned to recruit a total of 47 participants. According to the recruitment process, 27 students were enrolled in the experimental group and 20 in the control group. As no participants dropped out during the pretest, intervention, or posttest phases, data from all participants were included in the final analysis.

##### 2) Measurements

After selecting the measurement instruments, the researchers obtained permission for their use from the original authors.

###### (1) Digital sexual violence prevention knowledge

In this study, 10 knowledge items developed by Kim and Kang [21] were initially considered. These items assessed understanding of the concept, causes, and occurrence of sexual violence, potential sexual violence situations, difficulties and complications associated with sexual violence, and methods for seeking help following sexual violence incidents. Of these, seven items were modified and supplemented to align with the content of digital sexual violence and were used to measure digital sexual violence prevention knowledge. The appropriateness of the revised items was confirmed through expert consultation with one nursing professor and two health teachers. Each item was scored as 1 point for a correct response and 0 points for an incorrect response, yielding a total possible score ranging from 0 to 7, with higher scores indicating greater knowledge of digital sexual violence prevention.

###### (2) Self-protection behavior

Self-protection behavior was assessed using six items developed by Kim and Kang [21] to evaluate behaviors and coping abilities for self-protection in various sexual violence situations. Among these items, three were modified and supplemented to correspond specifically to digital sexual violence contexts and were used to measure self-protection behavior. The appropriateness of the revised items was confirmed through consultation with one nursing professor and two health teachers. Each item was scored as 1 point for a correct response and 0 points for an incorrect response, resulting in a total score ranging from 0 to 3, with higher scores indicating stronger self-protection behaviors related to digital sexual violence.

###### (3) Digital citizenship

In this study, digital citizenship was measured using 15 items on a 5-point Likert scale that were employed in the digital sexual violence prevention education study conducted by Kim [14]. The instrument comprised four subdomains: respect and consideration (five items), digital citizenship (three items), bystander self-efficacy (four items), and understanding of knowledge and response methods (three items). Response options ranged from 1 point (“not at all”) to 5 points (“very much so”). Scores for each subdomain were calculated as the sum of the corresponding items, with higher scores indicating higher levels within each subdomain. The validity of the instrument was confirmed at the time of its development, and Cronbach’s α values for reliability were reported as .77–.86 at pretest and .89–.94 at posttest [14]. In the present study, subdomain reliability ranged from .51 to .72 at pretest and from .53 to .82 at posttest.

###### (4) General characteristics

Participants’ gender, SNS use experience (including usage status, duration, frequency, and time), and sources of knowledge related to digital sexual violence prevention were assessed. Class numbers were collected to enable matching of pre- and post-survey questionnaires. After study completion, class numbers were replaced with unique identification codes, and the data were processed in a de-identified form.

##### 3) Data collection

The pre-survey was conducted using a questionnaire method in both groups between April 17 and April 21, 2024. The digital sexual violence prevention education program was implemented by the first researcher for two classes in the experimental group once per week over 4 weeks, from April 24 to May 24, 2024. The program consisted of four sessions, each lasting 40 minutes, and was delivered during regular health education class time (Table 1). During the first session, students were guided to understand the overarching concept of digital media and to present the types of digital media they commonly used, along with their characteristics. The second session employed role-playing activities to introduce the concepts of citizenship and respect for others within a digital society. The third session addressed digital sexual violence in a comprehensive manner. In the fourth session, students were educated on practical strategies for preventing digital sexual violence and on the role of a bystander when digital sexual violence situations occur. In contrast, the control group received four topics of general health education—obesity prevention, eye health, dental health, and infection prevention—none of which were expected to influence the outcomes of the educational intervention examined in this study. Because both the experimental and control groups were drawn from the same grade level, there was a potential risk of diffusion of the intervention content from the experimental group to the control group. To minimize this possibility, students in the experimental group were instructed in advance not to share any information learned during the program with students in the control group.

To evaluate program effectiveness, a post-survey was conducted using the questionnaire method in both groups on May 25–26, 2024. As no participants withdrew during the study period, pre- and post-survey data collected from all enrolled participants were included in the analysis. Because the first researcher conducted the study within the school setting, the educational program was provided to all students in the experimental classes to ensure students’ right to education, even if individual students or parents did not consent to research participation. After study completion, the same four-session digital sexual violence prevention education program was also provided to students in the control group to ensure equitable learning opportunities.

##### 4) Data analysis

Data were analyzed using SPSS version 26.0 to evaluate the effects of the prevention education program, and the level of statistical significance was set at 0.05. General characteristics of the experimental and control groups were summarized using frequencies, percentages, and multiple-response analyses. Homogeneity between the experimental and control groups with respect to general characteristics and dependent variables measured at pretest was assessed using the chi-square test. For hypothesis testing, between-group differences in posttest digital sexual violence prevention knowledge, self-protection behavior, and the four subdomains of digital citizenship were analyzed using the one-tailed independent t-test.

## Results

### Comparison of general characteristics and dependent variables between the experimental group and control group: homogeneity test

Regarding gender distribution, males accounted for 55.6% of the experimental group and 40.0% of the control group. Experience with SNS use was reported by 81.5% of participants in the experimental group and 85.0% in the control group. SNS use for 2 years or longer was reported by 68.2% of the experimental group and 58.8% of the control group. Daily SNS use was reported by 54.5% of the experimental group and 70.6% of the control group, while SNS use for less than 30 minutes to less than 2 hours was reported by 72.7% of the experimental group and 76.4% of the control group. In addition, the primary source of knowledge regarding sexual violence prevention was school for the majority of participants (92.6% in the experimental group and 90.0% in the control group). Homogeneity testing of general characteristics indicated no statistically significant differences between the two groups (Table 2).

Before the intervention, digital sexual violence prevention knowledge scores were high in both groups, with a mean score of 5.96 (±1.25) in the experimental group and 5.85 (±1.03) in the control group, and no statistically significant difference was observed between groups (t=0.32, *p*=.745). Self-protection behavior scores related to digital sexual violence, based on a maximum score of 3 points, were also high in both groups, with mean scores of 2.70 (±0.54) in the experimental group and 2.80 (±0.52) in the control group, and no statistically significant difference was identified (t=−0.61, *p*=.544). Comparisons of the four subdomains of digital citizenship between groups further indicated no statistically significant differences (Table 2). Specifically, in the respect and consideration domain, mean scores were 19.48 in the experimental group and 19.05 in the control group out of a possible 25 points, indicating a high level. In the digital citizenship domain, mean scores were 10.55 in the experimental group and 9.80 in the control group out of 15 points, reflecting a moderate level. In the bystander self-efficacy domain, mean scores were 13.85 in the experimental group and 13.15 in the control group out of 20 points, also indicating a moderate level. In the knowledge and response domain, mean scores were 10.07 in the experimental group and 10.60 in the control group out of 15 points, corresponding to a moderate level.

### Hypothesis verification

Regarding hypothesis 1, following the intervention, digital sexual violence prevention knowledge scores were 6.81 (±0.48) in the experimental group and 6.10 (±0.85) in the control group, demonstrating a statistically significant difference between groups (t=3.37, *p*<.001). Accordingly, hypothesis 1, which proposed that students who received the education program would demonstrate higher prevention knowledge than those who did not, was supported.

Regarding hypothesis 2, post-intervention self-protection behavior scores were 2.92 (±0.26) in the experimental group and 2.75 (±0.44) in the control group, and this difference was not statistically significant. Therefore, hypothesis 2 was not supported.

Regarding hypothesis 3, among the four subdomains of digital citizenship assessed after the intervention, the bystander self-efficacy domain showed a statistically significant difference between groups, with a mean score of 16.59 (±2.45) in the experimental group and 15.20 (±2.83) in the control group (t=1.79, *p*=.039). In contrast, although the experimental group demonstrated slightly higher mean scores than the control group in the remaining three subdomains, these differences were not statistically significant. Thus, hypothesis 3, which proposed that students who received the education program would demonstrate higher awareness of bystander self-efficacy within digital citizenship, was partially supported (Table 3).

## Discussion

This study developed a digital sexual violence prevention education program for fifth-grade students and applied the program over a 4-week period using a nonequivalent control group quasi-experimental design. The results showed that students who participated in the education demonstrated significantly higher digital sexual violence prevention knowledge scores and higher scores for bystander self-efficacy, a subdomain of digital citizenship, compared with the control group. Accordingly, two of the three research hypotheses were supported. Based on these findings, the outcomes of the program development and its application are discussed below.

When compared with the two-session education program developed by the AHA Seoul Municipal Youth Sex Culture Center for fifth–sixth-grade elementary school students, which focused primarily on enhancing digital citizenship [13], the present program targeted fifth-grade students, a similar population of upper-grade elementary school students. However, this program differed in that it expanded prevention education into an integrated and comprehensive format that simultaneously aimed to improve digital sexual violence prevention knowledge, self-protection behaviors, and digital citizenship, and increased the number of sessions to four. In addition, based on evidence indicating that upper-grade elementary school students are particularly vulnerable to digital sexual violence [5,6], this study developed and implemented the program within the school setting using health education content tailored to students’ developmental levels while securing sufficient instructional time. These characteristics distinguish the present study from previous research. Notably, because the program was designed as part of regular school health education and provided session-specific instructional materials along with diverse teaching–learning methods across four sessions, it is readily applicable in real-world school settings and allows for the gradual achievement of learning outcomes.

In this study, digital sexual violence prevention knowledge was significantly higher in the experimental group than in the control group following completion of the four-session program, thereby confirming the educational effect of the intervention. In particular, compared with the control group, the experimental group demonstrated higher correct response rates for items assessing the causes of digital sexual violence and the identification of situations that constitute digital sexual violence. This outcome is likely attributable to the program’s detailed coverage of the concepts, types, and real-world examples of digital sexual violence. These findings are consistent with previous studies in which, although no separate knowledge assessment tool was used, scores in the knowledge and response subdomain of digital citizenship increased after participation in a two-session digital sexual violence prevention education program among fifth–sixth-grade elementary school students [13], and similar improvements were observed when analyses were restricted to fifth-grade students alone [14]. Moreover, the present results align with a study reporting improvements in sexual knowledge following an eight-session digital sexual violence prevention education program (45 minutes per session) conducted among students with intellectual disabilities. Although their chronological ages ranged from 15 to 18 years, they were enrolled in the eighth grade of middle school due to their disabilities [22].

In contrast, the education program did not demonstrate a significant effect on self-protection behavior scores. This finding differs from review evidence suggesting that sexual violence prevention education programs for elementary school students can enhance not only knowledge but also children’s capacity to practice appropriate preventive behaviors [17]. One possible explanation for the lack of observed effect in this study is that self-protection behavior scores in both groups were already close to the maximum score at baseline, ranging from 2.7 to 2.8 out of 3 points, indicating a ceiling effect. As a result, even though scores in the experimental group increased slightly after the intervention, no statistically significant difference could be detected in comparison with the control group. In addition, because outcomes were evaluated immediately after the four-week education period, there may have been insufficient time for improved awareness of self-protective behaviors to translate into actual behavioral practice. This interpretation is consistent with prior review findings indicating that while knowledge related to sexual violence can be acquired over a short period, gender sensitivity and sexual attitudes—such as recognizing discrimination and inequality related to sex—are more difficult to develop in the short term [18]. Future research should therefore incorporate longer-term follow-up assessments to examine whether educational interventions lead to sustained and meaningful changes in self-protective behaviors.

Regarding digital citizenship, a significant effect of the education program was observed only in the bystander self-efficacy subdomain, with the experimental group scoring higher than the control group. This is different from previous studies in which all four subdomains of digital citizenship showed posttest improvements compared with pretest levels. These include studies analyzing only fifth-grade students [14] as well as studies including both fifth- and sixth-grade students [13] who participated in two-session digital sexual violence prevention education programs, and a study reporting posttest improvements in digital citizenship scores among fifth–sixth-grade elementary school students after a six-session digital sexual crime prevention education program [23]. The preceding studies [13,14,23] appear to have demonstrated immediate improvements in outcome indicators because they focused specifically on enhancing digital citizenship among upper-grade elementary school students. In contrast, the present study provided a comprehensive education program aimed at improving digital sexual violence prevention knowledge, self-protection behaviors, and digital citizenship simultaneously; therefore, temporal constraints may have limited the ability to observe immediate post-intervention effects on digital citizenship. Future strategies to enhance educational effectiveness should consider allocating practice time for situations requiring digital sexual violence prevention, incorporating experiential activities, demonstrations, and discussion processes related to respect and consideration, citizenship development, bystander self-efficacy, and knowledge and response skills, and evaluating the long-term effects of education with appropriate time intervals.

The significant effect observed in the bystander self-efficacy subdomain of digital citizenship in this study is similar to findings from a previous study conducted in Hawaii, USA, in which school-based sexual violence prevention education resulted in higher awareness of bystander intervention among high school students compared with a control group [24]. This suggests that educational content designed to enhance confidence in intervening as a bystander in sexual violence situations can promote not only strengthened bystander behaviors but also improved knowledge and attitudes related to sexual violence prevention and response. Accordingly, the finding that the present digital sexual violence prevention education program improved students’ active participation as bystanders in digital environments indicates that such programs may partially contribute to enhancing digital citizenship through school-based health education.

Digital citizenship education content should include digital ethics [9], defined as the attitude of behaving responsibly and ethically in digital environments, as well as critical literacy, which involves evaluating the authenticity of online information and selecting trustworthy sources, and productive literacy, which encompasses information creation skills and critical engagement with self-generated content [25]. However, in school settings, digital citizenship education is currently delivered only within a limited number of subjects, such as social studies, Korean language, and ethics. Moreover, digital sexual violence prevention education is often implemented as a component of subject-based instruction or sex education and is frequently delivered over short timeframes, leading to ongoing concerns regarding its effectiveness. Consequently, there is a need for educational approaches that can be integrated across both curricular and extracurricular domains [26]. To address this need, schools should secure instructional time separate from existing sex education classes and increase overall instructional hours to provide systematic education tailored to students’ developmental levels, including gender sensitivity education, digital citizenship, prevention of digital sexual violence victimization and perpetration, and victim protection education. In this context, it is anticipated that the digital sexual violence prevention education program developed in this study—focused on prevention knowledge, self-protection behavior, and digital citizenship—along with its instructional strategies, can contribute to strengthening the overall quality and effectiveness of sexual violence and digital sexual violence prevention education.

During the conduct of this study, several limitations were encountered due to the inclusion of elementary school children, a vulnerable population. Specifically, challenges arose in obtaining consent from both children and their parents, as well as in recruiting participants, owing to parents’ limited awareness of digital sexual violence prevention education and concerns regarding personal information exposure. Additionally, although homogeneity between the experimental and control groups was achieved through random assignment among fifth-grade students within a single elementary school, the fact that both groups were drawn from the same grade and located in close physical proximity raises the possibility that intervention effects may have diffused through informal communication between groups. Accordingly, future research should employ experimental designs involving multiple schools with comparable conditions, secure sufficiently large sample sizes, and extend evaluation periods in order to assess both the short-term effects of prevention education on knowledge and the mid-term effects on behavioral and digital citizenship outcomes.

In conclusion, this study developed a digital sexual violence prevention education program for upper-grade elementary school students and partially demonstrated the program’s effectiveness through an experimental research design that included a control group. To address limitations in school settings where digital sexual violence prevention education is often delivered only formally as part of sex education, this study is meaningful in that a school health teacher developed a four-session education program centered on comprehensive knowledge of digital sexual violence, prevention and response strategies, gender sensitivity, and digital citizenship. In addition, the study is significant in that educational effects were empirically verified and practical outcomes were achieved through a four-session health education program led by a school health teacher. Accordingly, it is proposed that this digital sexual violence prevention education program for upper-grade elementary school students be modified and supplemented to better align with school contexts and utilized as formal educational content. Future research should employ more rigorous experimental designs with larger sample sizes to evaluate the short- to mid-term effects of such educational interventions.

## Figures and Tables

**Table 1.** Detailed program of digital sexual violence prevention education for fifth-grade students

Session	Area	Subject	Learning objectives	Activity details	Education methods and media	Time (min)
1	Etiquette in the digital environment	Become friends with digital media	• Understand the characteristics of digital media, and based on this, recognize that it is easy to hurt others in the digital media world.	• Understanding the concept and considering the characteristics of digital media	Lecture, video, game, discussion, activity sheet	40
			• Can explain desirable norms to be observed in digital environments and learning communities.	• Understanding anonymity and dissemination through the photo passing game		
				• Understanding digital etiquette concepts and creating digital self-governance rules		
2	Citizen awareness of participating in creating a gender-equal digital environment	Living as a citizen of a digital society	• Can explain the concept of a citizen in a digital society.	• Understanding the concept of digital society and citizenship	Lecture, role play, picture, video, activity sheet	40
			• Can talk about the importance of using digital media while respecting others.	• Citizenship in a digital society: Respect for others		
				• Non-consensual photo sharing and digital citizenship		
				• Using media with respect for others		
3	Understanding digital sexual violence	Understand knowledge and response methods to digital sexual violence	• Can explain the various types of digital sexual violence that elementary school students may experience.	• Sharing indirect experiences about digital sexual violence	Lecture, video, card webtoon, creating Pop Grip	40
			• Tips for responding to digital sexual violence crises can be explained and put into practice.	• Understanding the concept and types of digital sexual violence		
				• Knowing how to respond to digital sexual violence		
				• Creating one’s own PopSocket (phone grip)		
4	Practice prevention of digital sexual violence	Role as an active bystander when sexual violence occurs in a digital environment	• Can explain what you need to be careful about when using media in your daily life to prevent digital sexual violence.	• Being familiar with cases of digital sexual violence that frequently occur in digital media environments	Lecture, activity sheet, making wish tree	40
			• Talk about what we can do to prevent digital sexual violence.	• Online grooming		
				• Finding out how to prevent damage		
				• Making a wish tree in our class		
				• Discussing what to do to prevent digital sexual violence		

**Table 2.** Homogeneity test of participants’ characteristics and variables between the experimental and control groups (N=47)

Variable	Category	n (%) or mean±SD		*χ*^2^ / t	*p*
		Experimental group (n=27)	Control group (n=20)		
Sex	Male	15 (55.6)	8 (40.0)	1.11	.292
	Female	12 (44.4)	12 (60.0)		
Experience in using SNS	Yes	22 (81.5)	17 (85.0)	1.000	–
	No	5 (18.5)	3 (15.0)		
SNS users only	Duration				
	<6 months	3 (13.6)	5 (29.4)	1.72^†^	.725
	≥6 to <12 months	2 (9.1)	1 (5.9)		
	≥1 to <2 years	2 (9.1)	1 (5.9)		
	≥2 years	15 (68.2)	10 (58.8)		
	Frequency (times/week)				
	1–2	2 (9.1)	1 (5.9)	1.22^†^	.814
	3–4	6 (27.3)	3 (17.6)		
	5–6	2 (9.1)	1 (5.9)		
	Every day	12 (54.5)	12 (70.6)		
	Usage time				
	<30 minutes	5 (22.7)	4 (23.5)	2.85^†^	.653
	≥30 to < 1 hour	5 (22.7)	5 (29.4)		
	≥1 to <2 hours	6 (27.3)	4 (23.5)		
	≥2 to <3 hours	3 (13.6)	4 (23.5)		
	≥4 hours	3 (13.6)	0 (0)		
Sources of sexual knowledge (multiple responses)	School	25 (92.6)	18 (90.0)	–	
	Family and friends	7 (25.9)	15 (75.0)		
	Mass media (news, TV, internet, books)	7 (25.9)	14 (70.0)		
Prevention knowledge	Possible range: 0–7	5.96±1.25	5.85±1.03	0.32	.745
Self-protection behavior	Possible range: 0–3	2.70±0.54	2.80±0.52	−0.61	.544
Digital citizenship	Respect and consideration (5–25)	19.48±3.60	19.05±3.60	0.41	.678
	Digital citizenship (3–15)	10.55±2.70	9.80±2.74	0.94	.352
Bystander self-efficacy	4–20	13.85±2.82	13.15±2.97	0.91	.365
Knowledge/response	3–15	10.07±2.26	10.60±2.54	−0.74	.495

SNS: Social networking service.

† Fisher exact test.

**Table 3.** Comparison of study variables between the two groups at posttest (N=47)

Variable	Mean±SD		t	*p*
	Experimental group (n=27)	Control group (n=20)		
Prevention knowledge	6.81±0.48	6.10±0.85	3.37	.001
Self-protection behavior	2.92±0.26	2.75±0.44	1.57	.063
Digital citizenship				
Respect and consideration	21.88±3.36	21.40±3.13	0.55	.615
Digital citizenship	11.33±2.27	11.50±1.98	–0.26	.794
Bystander self-efficacy	16.59±2.45	15.20±2.83	1.79	.039
Knowledge/response	11.51±2.19	11.25±2.65	0.38	.706
